# Pathological assessment of tumour beds in breast excisions post‐neoadjuvant therapy: a retrospective cohort study

**DOI:** 10.1111/his.70192

**Published:** 2026-06-10

**Authors:** Angela Cheng, Penelope Barnes, Daniel Rayson, Christopher Liwski, Gillian Bethune

**Affiliations:** ^1^ Department of Pathology Dalhousie University Halifax Nova Scotia Canada; ^2^ Division of Medical Oncology, Department of Medicine Dalhousie University Halifax Nova Scotia Canada

**Keywords:** breast cancer, chemotherapy, immunotherapy, laboratory, neoadjuvant therapy, pathology

## Abstract

**Aims:**

The pathological assessment of breast cancer resection specimens with neoadjuvant therapy (NAT) offers invaluable information for prognosis and further management. At times, these cases are difficult to pathologically evaluate, at least partly due to tumour bed obscuration after NAT. This study aimed to compare time and resource utilization by surgical pathology laboratories when handling breast specimens treated with NAT against those without NAT. We secondarily aimed to identify features associated with tumour bed identifiability at gross assessment.

**Methods and results:**

In this retrospective, single‐institution study, we identified 241 breast resection specimens with systemic NAT between 2019 and 2025. A 1:1 non‐NAT control group was randomly selected. The NAT cohort had a higher median number of tissue blocks (20.0 versus 13.0, *P* < 0.001), as well as larger proportions requiring gross resampling (23.7% versus 7.1%, *P* < 0.001) and pathologist review (16.2% versus 4.6%, *P* < 0.001) compared with the control. The NAT cohort had a longer median pathology report turnaround time (17.0 days versus 15.0 days, *P* < 0.001). In NAT cases, pretreatment clinical stage was associated with macroscopic identifiability of the tumour bed.

**Conclusions:**

Breast cancer resection specimens with NAT required significantly more time and laboratory resources than similar specimens without NAT. As NAT becomes more frequent across breast and other cancer types, pathological assessment of the excisional specimens from these patients is expected to become more challenging and time‐consuming. By recognizing these trends early, healthcare systems can plan and allocate resources accordingly.

## Graphical abstract

Pathological assessment of post‐neoadjuvant treatment (NAT) breast specimens uses more laboratory resources compared with non‐NAT cases. The highest resource utilization (specimen resampling, number of tissue blocks submitted, pathologist review) occurred when the tumour bed is not grossly identifiable. Macroscopic tumour beds in breast specimens post‐NAT. (A) Identifiable tumour bed. (B) Indeterminate tumour bed. (C) Unidentifiable tumour bed.

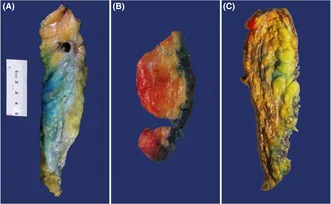

**Abbreviations**
CI: confidence interval
cT: clinical tumour stage
HER2: human epidermal growth factor receptor 2
IQR: interquartile range
NAT: neoadjuvant therapy
OR: odds ratio
pCR: pathological complete response
RCB: residual cancer burden
TAT: turnaround time
TN: triple‐negative

## Introduction

Uptake of neoadjuvant therapy (NAT) as part of breast cancer management has been steadily increasing in Canada and globally. While NAT was initially indicated only for patients with locally advanced and inflammatory breast cancer, this strategy has evolved into a standard of care for patients with human epidermal growth factor receptor‐2 (HER2) overexpressing or triple‐negative (TN) disease as a component of curative‐intent therapy, and to facilitate breast‐conserving surgery in some cases.^1^ This has had downstream effects on anatomical pathology resource utilization, as these post‐NAT specimens often require special attention for reporting of additional data elements.

**Figure 1:**
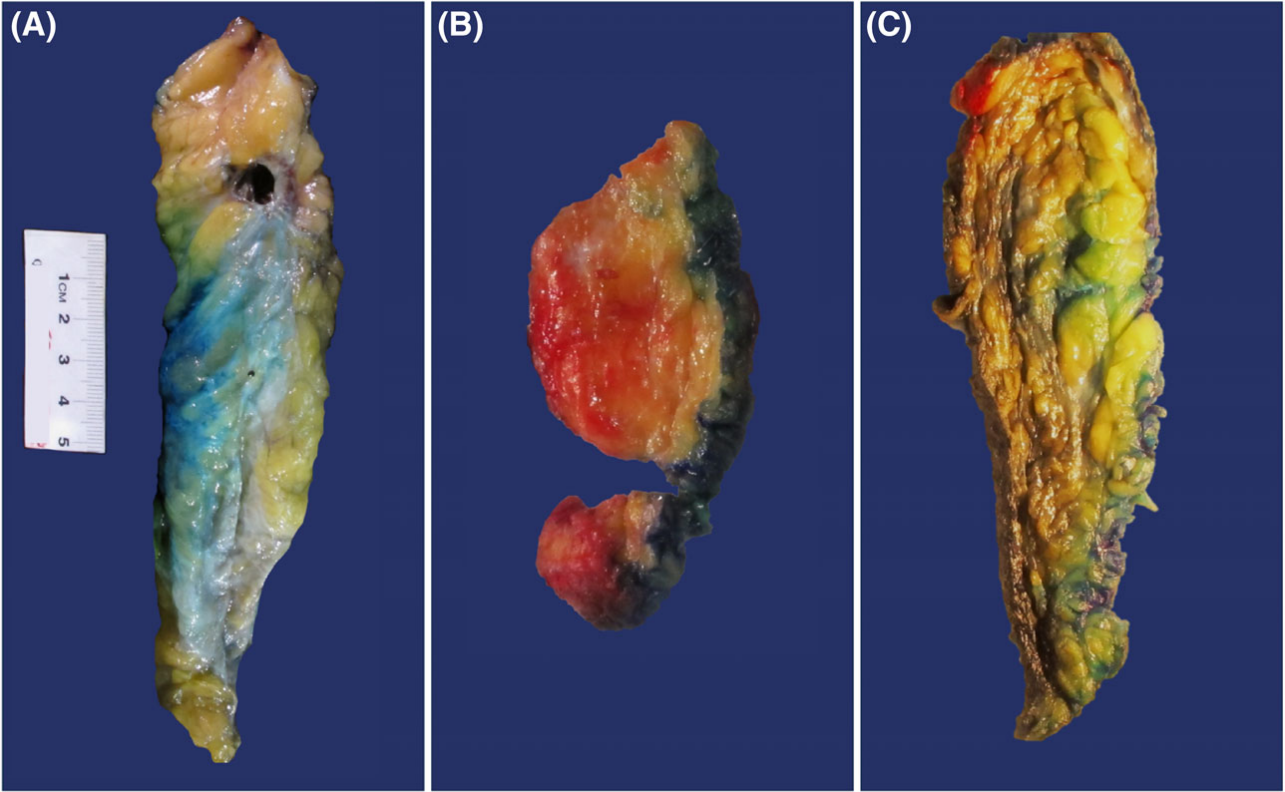
Macroscopic tumour beds in breast excisional specimens following neoadjuvant therapy. **(A)** Identifiable macroscopic tumour bed. **(B)** Indeterminate macroscopic tumour bed. **(C)** Unidentifiable macroscopic tumour bed.

In addition to pathological staging, the assessment of post‐NAT breast resection specimens requires the evaluation of treatment response. This relies on careful gross examination to identify the tissue containing the original pretreatment tumour, referred to as the tumour bed. The gross appearance of tumour beds varies, ranging from obvious residual tumour, fibrous scarring, or unremarkable tissue indistinguishable from the background. The latter may occur for various reasons, including profound responses to treatment, small primary tumours, and/or dense baseline breast tissue. As such, best laboratory practices include reviewing clinical and radiological information to guide gross specimen assessment (Figure 1).

**Figure 2:**
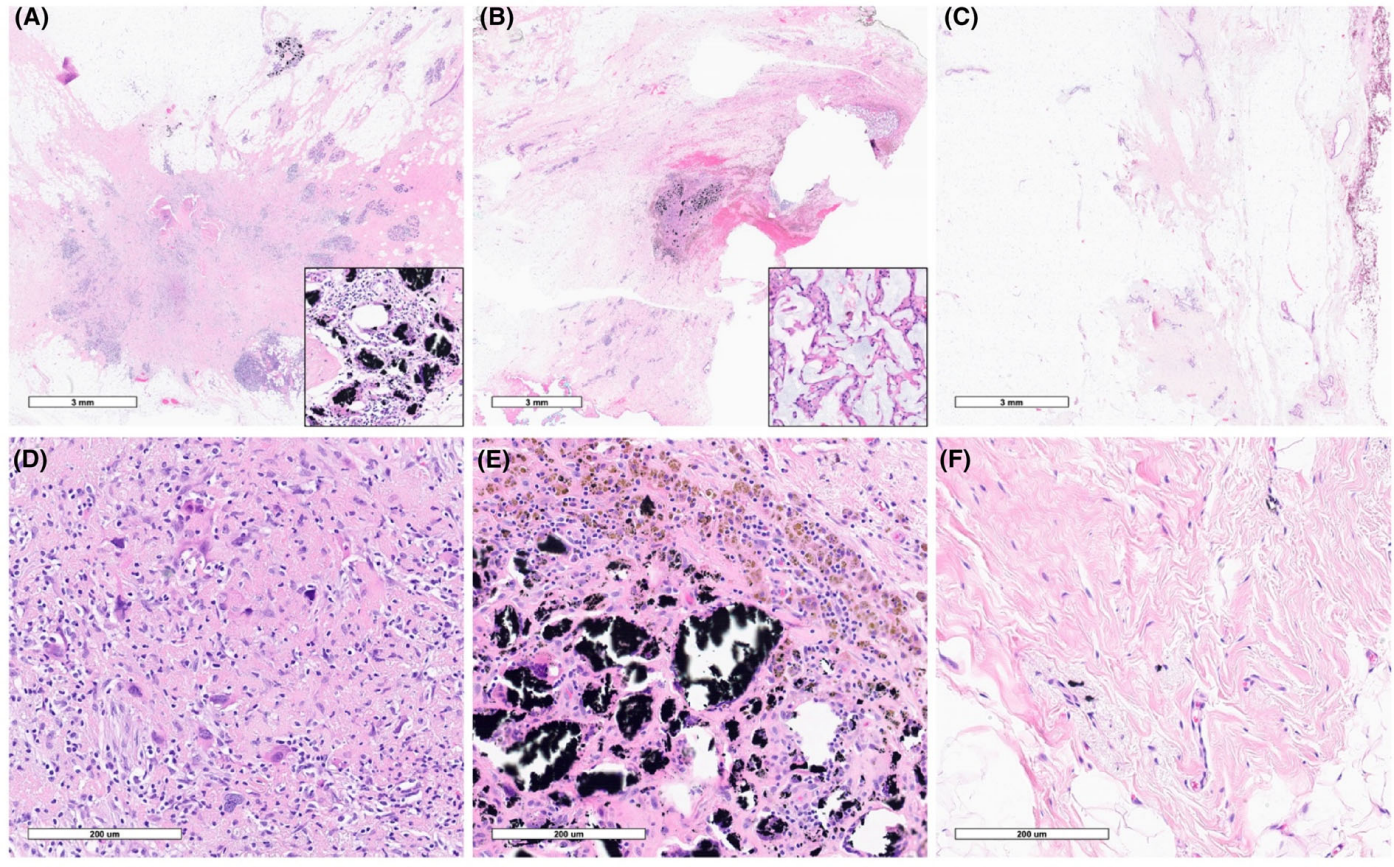
Representative histology of possible scenarios encountered in the pathological assessment of breast surgical specimens following receipt of neoadjuvant systemic therapy. **(A and D)** There is residual tumour. The cells have marked cytologic atypia, with surrounding fibrosis and inflammation, related to treatment effect. A carbon biopsy tract is present. **(B and E)** There is no residual tumour, but the tumour bed can be identified. There is a carbon biopsy tract, foamy macrophages, and hemosiderin‐laden macrophages. **(C and F)** There is neither a tumour nor strong evidence of a tumour bed. There is no significant inflammatory response or fibrosis. Scattered histiocytes with black pigment were noted, weakly suggestive of a carbon biopsy tract.

Specimen handling guidelines recommend representative sampling, including a complete cross‐section of the largest dimension of the tumour bed.^2^, ^3^, ^4^, ^5^ When the tumour bed cannot be identified grossly, the approximated site should be generously sampled proportionally to the pretreatment clinical tumour size, ideally with the aid of a core biopsy marker.^2^, ^4^ Accurate sampling of the tumour bed can be confirmed microscopically by the presence of residual tumour and/or evidence of treatment response, including foamy histiocytes, hemosiderin‐laden histiocytes, and fibrosis.^2^, ^3^, ^4^, ^5^ Insufficient tumour sampling requires reassessment and resampling of the gross specimen. In a small subset of cases, there is no residual tumour and only subtle treatment‐related changes despite exhaustive tissue examination, which can make it difficult to confirm tumour bed sampling with confidence (Figure 2). In small lumpectomies, this can raise concerns about the completeness of excision, and in mastectomies, can call into question the confidence of reporting a pathologic complete response (pCR). pCR has both prognostic and predictive value, and therefore it is crucial for pathologists to determine accurately the absence of residual disease. These situations present a clinicopathological dilemma; as preoperative therapies have become more common, effective, and specific, post‐therapy pathological assessment has become more labour‐intensive and challenging.

While the use of NAT has been increasing in the management of early breast cancer,^6^, ^7^, ^8^ less has been explored about the impact of this on hospital laboratories.

We hypothesize that breast resection specimens with NAT, particularly those with tumour beds that are difficult to grossly identify, require more laboratory resources and time for pathological diagnosis than those without preoperative therapy.

## Materials and Methods

### Study Population

Using our laboratory information system (Cerner Millennium), we retrospectively identified consecutive breast resection specimens collected at Nova Scotia Health between June 2019 and June 2025 containing a primary invasive breast carcinoma with at least one cycle of NAT. Only cases with available pretreatment biopsy pathology reports were included. The following cases were excluded: completion mastectomies, margin re‐excisions, and/or those from patients treated with palliative intent or endocrine therapy alone. In cases of multifocal or bilateral resections, the incidental foci (i.e., smaller or non‐NAT indicated) were excluded.

Demographic, radiological, and clinical information were collected from the original pathology reports and electronic medical records. Histological and biomarker subtypes were based on biopsy reporting. The TN subtype was further divided into those who received neoadjuvant immunotherapy and those who received neoadjuvant chemotherapy alone, to account for recent evolutions in NAT regimens for this population. Tumour bed gross features and treatment response were collected from the excisional pathology reports.

### Resource Utilization and Turnaround Time

For time and resource utilization comparisons, we identified 1677 consecutive breast resection cases for invasive primary breast cancer collected at our institution from the study period, after excluding the NAT cohort cases, microinvasive or *in situ* disease only, margin or cosmetic re‐excision, and prophylactic mastectomy cases. A 1:1 control group was randomly selected from this list, matched to the study cohort by excisional procedure type and collection date quartile.

For each study and control case, the number of tissue blocks submitted (excluding lymph node blocks), whether a breast pathologist was required for grossing review, and whether specimen resampling was required were extracted from the excisional pathology reports. Pathology report turnaround time (TAT) was calculated from the report collection and verification dates.

### Tumour Bed Gross Identifiability and Specimen Resampling

Gross tumour bed identifiability was determined from the synoptic gross description and coded as indeterminate, yes/identifiable, and no/unidentifiable. ‘Indeterminate’ was assigned when uncertainty was indicated (e.g., ‘possible’, ‘query’, ‘vague’). Descriptions of tumour beds without uncertainty were classified as ‘yes/identifiable’, while explicit absence (e.g., ‘no tumor bed identified’) were coded as ‘no/unidentifiable’.

Reasons for specimen resampling were extrapolated from the pathology report and coded as follows: (1) tumour bed not sampled on first grossing attempt, (2) tumour bed sampled on first grossing attempt, but more tissue needed for confirmation of complete response, (3) tumour bed sampled on first grossing attempt, but more tissue needed for characterization of the degree of partial response, and (4) non‐tumour bed concern.

### Statistical Analysis

Categorical variables were reported as frequencies, and continuous variables as medians with interquartile ranges (IQRs). Categorical outcomes were compared by the chi‐square (χ^2^) test. Continuous outcomes were compared by the Mann–Whitney *U* and Kruskal–Wallis tests. Post hoc pairwise comparisons were performed with Bonferroni correction. Univariate multinomial and univariate logistic regression models were used to determine predictive factors for macroscopic tumour bed identifiability and for the need for specimen resampling, respectively. Statistical analyses were performed using Stata software.

## Results

### Clinicopathological Characteristics

About 241 patients met our study criteria. Demographic, clinical, and pathological features are presented in Table 1. Of the 241 cases, the gross tumour bed was identifiable in 150 cases (62.2%), indeterminate in 47 cases (19.5%), and unidentifiable in 44 cases (18.3%).

**Table 1:** Clinicopathological features of breast surgical resection specimens containing invasive breast carcinoma following receipt of curative‐intent neoadjuvant systemic therapy (*N* = 241). Cases that required resampling are in the right column (*n* = 57)

	All cases (*N* = 241)	Resampled cases only (*n* = 57)
*Clinicopathological feature*		
Age (years)	53.0 (11.8)	52.8 (12.0)
≤40	38 (15.8%)	9 (15.8%)
>40 to ≤50	63 (26.1%)	18 (31.6%)
>50 to ≤60	64 (26.6%)	12 (21.1%)
>60	76 (31.5%)	18 (31.6%)
Clinical stage^a^	*N* = 233	*n* = 54
cT1	55 (23.6%)	16 (29.6%)
cT2	116 (49.8%)	25 (46.3%)
cT3	27 (11.6%)	4 (7.4%)
cT4	35 (15.0%)	9 (16.7%)
Histological subtypes		
Ductal	219 (90.9%)	54 (94.7%)
Lobular	13 (5.4%)	2 (3.5%)
Mixed	5 (2.1%)	1 (1.8%)
Metaplastic	4 (1.7%)	‐
Biomarker status		
HER2‐positive	93 (38.6%)	22 (38.6%)
TN, immunotherapy and chemotherapy	46 (19.1%)	11 (19.3%)
TN, chemotherapy only	50 (20.7%)	11 (19.3%)
HR‐positive, HER2‐negative	52 (21.6%)	13 (22.8%)
Neoadjuvant chemotherapy 100% complete	212 (88.0%)	53 (93.0%)
Surgery		
Lumpectomy	94 (39.0%)	16 (28.1%)
Mastectomy	147 (61.0%)	41 (71.9%)
Macroscopic tumour bed identifiability		
No/unidentifiable	44 (18.3%)	21 (36.8%)
Yes/identifiable	150 (62.2%)	8 (14.0%)
Indeterminate	47 (19.5%)	28 (49.1%)
Biopsy marker grossly identified	176 (73.0%)	36 (63.2%)
RCB class^a^	*N* = 239	*n* = 52
0	76 (31.8%)	25 (44.6%)
I	33 (13.8%)	9 (16.1%)
II	79 (33.1%)	20 (35.7%)
III	51 (21.3%)	2 (3.6%)
Reason for specimen resampling		
Search for missed tumour bed	‐	5 (8.8%)
Confirmation of complete response	‐	28 (49.1%)
Characterization of partial response	‐	20 (35.1%)
Non‐tumour bed concern	‐	4 (7.0%)

HER2, human epidermal growth factor receptor 2; HR, hormone receptor; TN, triple‐negative; RCB, Residual Cancer Burden.

^a^ Clinical stage and RCB class were inaccessible in eight and two cases, respectively.

About 57/241 cases required specimen resampling (23%). In most of these cases, the tumour bed was sampled during initial gross assessment but required resampling either to confirm a complete response (28/57, 49.1%) or to better assess the degree of partial response (20/57, 35.1%). Four cases were resampled for non‐tumour bed reasons (4/57, 7.0%), including lymph node or surgical margin assessment. In the remaining five resampled cases, the tumour bed was entirely missed on the initial gross assessment (8.8%); the tumour bed was found on the repeat gross assessment in one of these cases, but there was no definitive evidence of a tumour bed in the other four cases.

### Resource Utilization and Turnaround Time

Resource use and TAT comparisons of the NAT and control cases are given in Table 2. Resampling was more frequent in the NAT cohort than the control (23.7% versus 7.1%, *P* < 0.001). Similarly, pathologist grossing review occurred more often in the NAT group (16.2% versus 4.6%, *P* < 0.001). Median tissue submission was also higher in the NAT cohort than the control (20.0 blocks [IQR 14.0, 26.0] versus 13.0 blocks [IQR 10.0, 18.0], *P* < 0.001). Further, median report TAT was longer in the NAT cohort than the control cohort (17.0 days [IQR 13.0, 21.0] versus 15.0 days [IQR 13.0, 19.0], *P* < 0.001).

**Table 2:** Resource utilization and pathology report turnaround time for primary invasive breast carcinoma resection specimens with and without neoadjuvant systemic therapy (*N* = 482)

	Non‐NAT (*N* = 241)	NAT (*N* = 241)	*P*‐value
Specimen resampling^a^	17 (7.1%)	57 (23.7%)	<0.001
Required pathologist^a^	11 (4.6%)	39 (16.2%)	<0.001
Number of blocks^b^	13.0 (IQR 10.0, 18.0)	20.0 (IQR 14.0, 26.0)	<0.001
Turnaround time (days)^b^	15.0 (IQR 13.0, 19.0)	17.0 (IQR 13.0, 21.0)	<0.001

IQR, interquartile range; NAT, neoadjuvant therapy.

^a^ Chi‐square test.

^b^ Mann–Whitney *U*.

Workflow comparisons of the NAT cohort stratified by tumour bed macroscopic identifiability and control cases are given in Table 3. Pairwise comparisons are presented in Table S3. Higher resource utilization was driven by cases with grossly unidentifiable tumour beds. Compared with NAT cases with grossly identifiable tumour beds, those with grossly unidentifiable tumour beds had more frequent resampling (47.7% versus 18.7%, *P* < 0.001) and pathologist gross review (34.1% versus 8.7%, *P* < 0.001). Median tissue submission was higher in cases with unidentifiable tumour beds than in identifiable cases (25.5 blocks versus 16.0 blocks, *P* < 0.001). Difference in TAT was driven primarily by the unidentifiable tumour bed cases compared with the control cases (19.0 days [IQR 14.0, 21.5] versus 15.0 days [13.0, 19.0], *P* = 0.002). We did not detect differences in TAT between other comparisons.

**Table 3:** Resource utilization and pathology report turnaround time by degree of macroscopic tumour bed identifiability in resection specimens with neoadjuvant systemic therapy (*N* = 482)

	Non‐NAT	NAT by macroscopic tumour bed identifiability			
	Non‐NAT (*N* = 241)	Identified (*n* = 150)	Indeterminate (*n* = 47)	Not identified (*n* = 44)	*P*‐value
Specimen resampling^a^	17 (7.1%)	28 (18.7%)	8 (17.0%)	21 (47.7%)	<0.001
Required pathologist^a^	11 (4.6%)	13 (8.7%)	11 (23.4%)	15 (34.1%)	<0.001
Number of blocks^b^	13.0 (IQR 10.0, 18.0)	16.0 (IQR 12.0, 22.0)	26.0 (IQR 18.0, 33.0)	25.5 (IQR 19.0, 32.0)	<0.001
Turnaround time (days)^b^	15.0 (IQR 13.0, 19.0)	17.0 (IQR 13.0, 20.0)	18.0 (IQR 14.0, 20.0)	19.0 (IQR 14.0, 21.5)	0.002

Bonferroni correction is applied for assigning significance (alpha = 0.01).

IQR, interquartile range; NAT, neoadjuvant therapy.

^a^ Chi‐square test.

^b^ Kruskal‐Wallis.

### Predictive Factors of Macroscopic Tumour Bed Identifiability and the Need for Specimen Resampling

Predictive and association factors of tumour bed gross identifiability are given in Table S2. Pretreatment clinical tumour (cT) stage was associated with post‐NAT tumour bed macroscopic identifiability, driven by a difference between cT2 and cT1 (*P* = 0.0297). Compared with cT1, cT2 had lower odds of having a grossly unidentifiable than identifiable tumour bed (OR 0.30, 95% CI: 0.13, 0.68). RCB class was also associated with tumour bed identifiability (*P* < 0.001), driven by comparisons between extremes. Compared with RCB III, RCB 0 was associated with a much higher odds of having an unidentifiable tumour bed post‐NAT than an identifiable one (OR 18.4, 95% CI: 4.07, 83.60). A significant difference was not detected between comparisons of smaller increments, such as between RCB II and RCB III.

Predictive and association factors of the need for specimen resampling are given in Table S3. When the tumour bed was not grossly identifiable, there was a nearly fourfold increase in the odds of resampling being performed (OR 3.98, 95% CI: 1.94–8.17, *P* = 0.0004). There were trends toward higher odds of resampling with mastectomy than with lumpectomy (OR 1.89, 95% CI: 0.99–3.60, *P* = 0.0549) and lower odds of resampling with the presence of a biopsy marker (OR 0.54, 95% CI: 0.29–1.02, *P* = 0.0566), although these differences were not statistically significant.

## Discussion

In this study, we explored the impact of presurgical systemic therapy on anatomical pathology laboratory workflow when processing and reporting post‐NAT breast resection specimens.

We found that at our institution, breast resection specimens with presurgical systemic therapy required more laboratory resources than those without NAT. When stratified by tumour bed gross identifiability, the unidentifiable cases consistently used more resources than the identifiable cases and the non‐NAT cases. We did not observe a directly proportional relationship between resource utilization and the degree of macroscopic tumour bed identifiability. The indeterminate subgroup was like the identifiable subgroup with lower requirements for specimen resampling, while also being like the unidentifiable subgroup with higher requirements for pathologist review and amounts of tissue submission. Considering tissue submission further, the cost of producing one H&E slide at our institution is $36 CAD, excluding the time of pathologists and pathologist assistants for resampling and re‐examining specimens. This equates to an additional cost of approximately $250 CAD for NAT cases than non‐NAT cases, when comparing median tissue blocks (20 versus 13). The cost difference climbs to approximately $470 CAD when comparing tissue medians in grossly unidentifiable/indeterminate tumour bed cases to non‐NAT cases (26 versus 13).

Median report TAT was significantly longer in the NAT cohort than in the non‐NAT cohort, driven by the difference between the identifiable tumour bed cohort and the non‐NAT cohort. While there was no significant difference in TAT between the NAT cases when split by macroscopic tumour bed identifiability, there was an upwards trend from identifiable to unidentifiable. We hypothesize that TAT may be multifactorial involving grossing factors, histological review, and ancillary testing.

We then searched for clinicopathological features predictive of tumour bed gross identifiability. We initially theorized that molecular subtype would be related to macroscopic tumour bed discernibility, as HER2‐positive and TN tumours have had higher rates reported of pCR than hormone receptor (HR) positive/HER2‐negative tumours.^9^, ^10^, ^11^, ^12^ However, of the association factors we analyzed, only cT stage and RCB class held significance. Compared with cT1, there was a trend for higher stages to be more likely to have grossly identifiable tumour beds than unidentifiable tumour beds. As well, lower RCB classes were associated with more difficult tumour bed gross identifiability. We did not detect significant differences among comparisons between consecutive RCB classes, such as RCB II and III, or RCB 0 and I. As we know, post‐NAT tumour beds often contain fibrous scarring. This fibrosis likely dominates the macroscopic impression of the tumour bed, limiting the ability to grossly discern between small differences in residual tumour.

Tumour bed gross identifiability was a significant predictor of specimen resampling, with unidentifiable gross tumour beds associated with fourfold higher odds of specimen resampling than identifiable tumour beds. This suggests that uncertainty in gross sampling adequacy increases the likelihood of specimen resampling, which appears to be regardless of treatment response. Although not statistically significant, mastectomy specimens and cases without a discernible biopsy marker trended toward increased resampling, compared with lumpectomy specimens and cases with a biopsy marker, respectively. Mastectomy specimens may be overrepresented in the cases requiring resampling due to larger tissue volume limiting upfront *in toto* submission. Biopsy site markers (carbon pigment, metal coils) appeared protective against resampling, probably by increasing confidence in tumour bed sampling; however, they were not identified in over one‐quarter of cases, likely reflecting dislodgement or difficulty in detection, particularly in larger specimens.

Four of our cases had no definitive macroscopic or microscopic evidence of a tumour bed. As these situations can represent two entirely different scenarios (i.e., no residual tumour with an extremely subtle tumour bed or an incompletely excised residual tumour/tumour bed), substantial efforts must be made by the pathologist to distinguish between them to convey accurate prognostic and predictive information to the clinician and patient. Despite resampling and exhaustive tissue submission in these four cases, conclusive tumour bed features were not identified. After multidisciplinary discussion, these patients were followed with increased clinical surveillance and managed as cases with pCR with no clinical recurrences to date.

This study is the first attempt, to the best of our knowledge, at quantifying the resources used by hospital laboratories to process and report surgical specimens with presurgical therapy, bringing attention to an important topic. We showed quantitative evidence of the impacts of advancing presurgical therapy on hospital laboratory services. The strengths of our study include novelty and overall sample size. This study has several limitations. The control group was selected to reflect the non‐NAT caseload; however, as NAT cases inherently involve larger tumours, tissue submission may reflect tumour size rather than NAT‐related complexity alone. The retrospective design also limits reliability, particularly in coding tumour bed identifiability, which is subject to variability in grossing technique and terminology. Finally, the use of univariate analyses limits interpretability; multivariate models were attempted, but unsuccessful due to poor model fit with small, stratified sample sizes. Several factors related to macroscopic tumour bed identifiability also remain to be explored, including the quality and composition of background breast tissue, and reactive/immunological host response. It is plausible that variations in the latter factor may explain variability in macroscopic tumour bed identifiability.

We have entered a fascinating era of cancer management, where the degree of therapeutic response can be so significant that it becomes challenging to detect any sign of the original tumour. Although beneficial to our patients, this situation poses a significant strain on laboratory services, which may intensify as therapies evolve. By predicting these trends, we may be better prepared to meet changing resource demands in healthcare.

## Author contributions

AC collected the data and wrote the manuscript. GB and PB designed the research study. DR provided clinical insight for the study design. CL conducted a literature search and obtained ethics approval for the study. All authors contributed to editing the final manuscript.

## Funding information

No funding was received for this study.

## Conflicts of interest

The authors have no relevant conflicts of interest.

## Patient consent statement

A waiver of consent was obtained for this study.

## Supporting information


**Table S1.** Post hoc pairwise tests for resource utilization and pathology report turnaround time by degree of macroscopic tumour bed identifiability in resection specimens with neoadjuvant systemic therapy (*N* = 482).
**Table S2**. Univariate multinomial models for predictive and associative factors related to macroscopic tumour bed identifiability.
**Table S3**. Univariate logistic regression models for specimen resampling.

## Data Availability

The data that support the findings of this study are available from the corresponding author upon reasonable request.
