# Supplementary material for: Pathological assessment of tumour beds in breast excisions post‐neoadjuvant therapy: a retrospective cohort study
Source: Histopathology. 2026 Jun 10;89(4):650–7. doi: 10.1111/his.70192 (PMC13551208; doi:10.1111/his.70192)
Supplement: Supplementary file 1 [file HIS-89-650-s001.docx]

**Supplementary Table 1.** Post hoc pairwise tests for resource utilization and pathology report turnaround time by degree of macroscopic tumor bed identifiability in resection specimens with neoadjuvant systemic therapy (*N*=482).

|  | N vs. Y | N vs. I | N vs.  non-NAT | Y vs. I | Y. vs  non-NAT | I. vs  non-NAT |
| --- | --- | --- | --- | --- | --- | --- |
| Specimen resampling* | **<0.001** | **0.002** | **<0.001** | 0.799 | **<0.001** | 0.026 |
| Required pathologist* | **<0.001** | 0.259 | **<0.001** | 0.007 | 0.100 | **<0.001** |
| Number of blocks** | **<0.001** | 0.861 | **<0.001** | **<0.001** | **<0.001** | **<0.001** |
| Turnaround time (days)** | 0.140 | 0.290 | **0.002** | 0.711 | 0.009 | 0.035 |

**chi-square test, **Mann-Whitney U*

*Bonferroni correction is applied for assigning significance (alpha = 0.0021)*

*Abbreviations: NAT, neoadjuvant therapy; Y, yes/identifiable; N, no/unidentifiable; I, indeterminate*

**Supplementary Table 2.** Univariate multinomial models for predictive and associative factors related to macroscopic tumor bed identifiability.

|  | *N* | Macroscopic tumor bed | Relative risk | 95% confidence interval | *p*-value |
| --- | --- | --- | --- | --- | --- |
| Age (per year) | 241 | No vs Yes | 1.00 | 0.97, 1.03 | 0.9769 |
|  |  | Possible vs Yes | 1.00 | 0.97, 1.03 |  |
| Age categories (years)  >40 to ≤50 vs ≤40 | 241 | No vs Yes | 0.77 | 0.27, 2.15 | 0.5077 |
|  |  | Possible vs Yes | 0.51 | 0.17, 1.55 |  |
| >50 to ≤60 vs. ≤40 |  | No vs Yes | 0.56 | 0.19, 1.71 |  |
|  |  | Possible vs Yes | 1.20 | 0.45, 3.22 |  |
| >60 vs. ≤40 |  | No vs Yes | 0.96 | 0.36, 2.57 |  |
|  |  | Possible vs Yes | 0.84 | 0.31, 2.29 |  |
| Pre-NAT clinical size (per mm) | 226 | No vs Yes | 0.99 | 0.97, 1.00 | 0.3299 |
|  |  | Possible vs Yes | 1.00 | 0.98, 1.01 |  |
| Pre-NAT clinical tumor stage  cT2 vs cT1 | 233 | No vs Yes | 0.30 | 0.13, 0.68 | 0.0297 |
|  |  | Possible vs Yes | 0.65 | 0.27, 1.56 |  |
| cT3 vs cT1 |  | No vs Yes | 0.43 | 0.14, 1.38 |  |
|  |  | Possible vs Yes | 0.44 | 0.11, 1.82 |  |
| cT4 vs cT1 |  | No vs Yes | 0.33 | 0.10, 1.13 |  |
|  |  | Possible vs Yes | 1.54 | 0.55, 4.32 |  |
| Biomarker subtype  HER2+ vs. HR+ | 241 | No vs Yes | 1.87 | 0.75, 4.64 | 0.1835 |
|  |  | Possible vs Yes | 0.91 | 0.37, 2.19 |  |
| TN-I vs. HR+ |  | No vs Yes | 1.24 | 0.42, 3.62 |  |
|  |  | Possible vs Yes | 0.70 | 0.24, 2.04 |  |
| TN-C vs. HR+ |  | No vs Yes | 0.36 | 0.09, 1.49 |  |
|  |  | Possible vs Yes | 1.15 | 0.45, 2.92 |  |
| Neoadjuvant chemotherapy 100% complete (yes vs. no) | 241 | No vs Yes | 1.06 | 0.37, 3.05 | 0.9789 |
|  |  | Possible vs Yes | 0.93 | 0.35, 2.50 |  |
| Mastectomy vs. lumpectomy | 241 | No vs Yes | 0.58 | 0.29, 1.14 | 0.2492 |
|  |  | Possible vs Yes | 1.02 | 0.52, 2.02 |  |
| Biopsy marker (yes vs. no) | 241 | No vs Yes | 0.59 | 0.28, 1.22 | 0.2593 |
|  |  | Possible vs Yes | 0.65 | 0.32, 1.33 |  |
| RCB Class  0 vs. III | 239 | No vs Yes | 18.4 | 4.07, 83.6 | <0.001 |
|  |  | Possible vs Yes | 3.25 | 1.25, 8.40 |  |
| I vs. III |  | No vs Yes | 6.47 | 1.19, 35.1 |  |
|  |  | Possible vs Yes | 2.16 | 0.70, 6.62 |  |
| II vs. III |  | No vs Yes | 3.18 | 0.65, 15.5 |  |
|  |  | Possible vs Yes | 1.06 | 0.40, 2.82 |  |

*Abbreviations: HER2+, human epidermal growth factor receptor 2-positive, HR+, hormone receptor-positive; NAT, neoadjuvant therapy; RCB, residual cancer burden; TN-I, triple-negative with immunotherapy; TN-C, triple-negative with chemotherapy only*

**Supplementary Table 3.** Univariate logistic regression models for specimen resampling.

|  | N | Odds ratio | 95% Confidence Interval | *p-*value |
| --- | --- | --- | --- | --- |
| Age (per year) | 241 | 1.00 | 0.97, 1.02 | *0.8897* |
| Age groups (years)  >40 to ≤50 vs. ≤40 | 241 | 1.29 | 0.51, 3.25 | 0.6413 |
| >50 to ≤60 vs. ≤40 |  | 0.74 | 0.28, 1.97 |  |
| >60 vs. ≤40 |  | 1.00 | 0.40, 2.50 |  |
| Pre-NAT clinical size (per mm) | 226 | 0.99 | 0.97, 1.00 | 0.1158 |
| Pre-NAT clinical tumor stage  cT2 vs. cT1 | 233 | 0.67 | 0.32, 1.39 | 0.4950 |
| cT3 vs. cT1 |  | 0.42 | 0.13, 1.42 |  |
| cT4 vs. cT1 |  | 0.84 | 0.32, 2.19 |  |
| Neoadjuvant chemotherapy 100% complete (yes vs. no) | 241 | 2.08 | 0.69, 6.26 | 0.1911 |
| Mastectomy vs. lumpectomy | 241 | 1.89 | 0.99, 3.60 | 0.0549 |
| Biopsy marker (yes vs. no) | 241 | 0.54 | 0.29, 1.02 | 0.0566 |
| Macroscopic tumor bed  Indeterminate vs. identifiable | 241 | 0.89 | 0.38, 2.12 | 0.0004 |
| Not identifiable vs. identifiable |  | 3.98 | 1.94, 8.17 |  |
| Biomarker subtype  HER2-positive vs. HR+ | 241 | 0.93 | 0.42, 2.05 | 0.9881 |
| TN-I vs. HR+ |  | 0.94 | 0.37, 2.37 |  |
| TN-C vs. HR+ |  | 0.85 | 0.34, 2.12 |  |

*Abbreviations: HER2+, human epidermal growth factor receptor 2-positive, HR+, hormone receptor-positive; NAT, neoadjuvant therapy; RCB, residual cancer burden; TN-I, triple-negative with immunotherapy; TN-C, triple-negative with chemotherapy only*
